# Special Issue “Genetic Engineering of Plants for Stress Tolerance”

**DOI:** 10.3390/ijms262211138

**Published:** 2025-11-18

**Authors:** Moxian Chen

**Affiliations:** State Key Laboratory of Green Pesticide, Key Laboratory of Green Pesticide and Agricultural Bioengineering, Ministry of Education, Research and Development Center for Fine Chemicals, Guizhou University, Guiyang 550025, China; cmx2009920734@gmail.com

## 1. Advances in Molecular Understanding of Plant Stress Tolerance

Plants are continuously exposed to diverse environmental challenges such as drought, salinity, and temperature extremes, which restrict their growth and yield potential. With the accelerating pace of climate change, improving stress tolerance in plants has become an urgent global priority. Genetic engineering, combined with high-throughput omics and synthetic biology, has transformed our ability to dissect the complex networks governing plant adaptation. Integrative omics approaches, spanning metabolomics, transcriptomics, and phosphoproteomics, have enabled a system-level understanding of stress responses. For example, analyses of maize have revealed transcriptional regulation and metabolic specialization in alkaloid biosynthesis and ureide metabolism that underlie their ecological adaptation [1]. Similarly, combined metabolomic and transcriptomic profiling of wheat during the reproductive stage under drought stress has identified amino acid metabolism, heat shock proteins, and transporter systems as key contributors to thermal tolerance [2]. Complementary phosphoproteomic studies in maize have demonstrated that phosphorylation events mediated by MAPK signaling play crucial roles in early heat stress responses [3]. These studies collectively emphasize that stress tolerance arises from intricate molecular coordination across transcriptional, metabolic, and post-translational levels. Moreover, recent discoveries highlight the critical role of alternative splicing in modulating plant developmental transitions under environmental stress. A striking example is the identification of VRF1 alternative splicing as a molecular switch that regulates stress-induced early flowering in Arabidopsis [4]. The study demonstrated that isoform ratios of VRF1 determine whether plants prioritize stress tolerance or reproductive escape, offering a new mechanistic insight into plant adaptation strategies.

## 2. Genetic Engineering, Synthetic Modules, and Regulatory Mechanisms

Alongside omics-driven insights, genetic engineering and synthetic biology have opened new avenues for developing crops with enhanced stress resilience. The creation of synthetic multi-gene modules exemplifies this progress. High expression of the class II *TPS* gene osTPS8 in rice significantly improved salinity tolerance by enhancing osmotic adjustment, activating antioxidant defense systems, and upregulating stress-related genes [5]. This demonstrates how synthetic modules integrating protective functions from extremophiles can be used to fortify plants against multiple stress factors simultaneously.

Genome-wide identification of transcription factor families has also advanced our understanding of regulatory networks involved in stress responses. The characterization of the TCP gene family in *Cenchrus fungigraminus* revealed genes responsive to drought and cold stress, with several members associated with growth and developmental regulation [6]. Beyond plant-specific mechanisms, cross-disciplinary studies have revealed parallels between plant stress adaptation and resistance mechanisms across biological systems. For instance, recent reviews on drug target discovery and membrane protein-mediated resistance provide valuable perspectives for plant biotechnology [7,8]. These works illustrate how convergent evolution and membrane-associated signaling contribute to adaptive resistance in organisms ranging from microbes to plants, highlighting shared molecular logic that can inspire the design of novel resistance-breaking strategies in crops. Moreover, alternative splicing has emerged as a vital post-transcriptional mechanism that fine-tunes gene expression during plant–fungal symbioses, balancing immune responses and beneficial interactions [9]. Together, these findings highlight that plant resilience depends not only on the activation of stress-related genes but also on sophisticated transcriptional and post-transcriptional regulation.

## 3. Evolutionary Insights and Future Perspectives

Exploring stress adaptation in naturally resilient species continues to provide valuable lessons for crop improvement. Investigations of high-altitude plants from the Qinghai–Tibet Plateau revealed that long-term adaptation to low temperatures, intense UV radiation, and nutrient-poor soils involves integrated physiological and morphological adjustments, including enhanced antioxidant activity and photosynthetic efficiency [10]. When viewed together with molecular insights such as VRF1-mediated flowering control and membrane protein-driven resistance evolution, it becomes clear that plants employ both evolutionary and engineered strategies to optimize survival under stress. These insights exemplify the evolutionary templates that modern biotechnology seeks to emulate through targeted engineering.

Collectively, the studies in this Special Issue provide a multidimensional perspective on plant stress tolerance, demonstrating that sustainable agricultural productivity depends on the integration of molecular, synthetic, and ecological approaches. Omics-based discoveries guide the identification of key pathways, while synthetic biology offers the tools to reprogram plants for enhanced performance under stress. Future research should focus on validating engineered traits under field conditions, where multiple stresses often occur concurrently, and on exploring developmental stage-specific and combined stress responses.

Integrating multi-omics data with ecological modeling will also be crucial for deploying stress-tolerant varieties in suitable environments. The convergence of these efforts will accelerate the creation of crops capable of maintaining productivity and stability under climate variability.

The guest editors express their sincere gratitude to all authors, reviewers, and the editorial team of the *International Journal of Molecular Sciences* for their invaluable contributions. Collectively, these papers mark an important step forward in applying molecular science and biotechnology to ensure agricultural sustainability in a changing world.
